# 

**Published:** 1895-02-09

**Authors:** 


**Notices of Births, Deaths, and Marriages** are inserted in this column at a charge of 2s. 6d. for an announcement not exceeding four lines.

**Notice to Advertisers and Subscribers.**

All Advertisements, Orders for Copies of The Hospital and Business Communications should be addressed to **The Manager, NOT TO THE EDITOR**, The Hospital, 428, Strand, London, W.C.

The Cost of Subscription, post free, is as follows:—Per week, 2½d.; per quarter, 2s. 8d.; per half-year, 5s. 3d.; per annum, 10s. 6d. Foreign:—Per Annum, 15s. 2d.; payable, in all cases, in advance.

### NOTICE TO CORRESPONDENTS.

All MS., Letters, Books for Review, and other matters intended for the Editor should be addressed The Editor, The Lodge, Porchester Square, London, W.

The Editor cannot undertake to return rejected MS., even when accompanied by stamped directed envelope.

**"Burdett's Hospital Annual," 1895,**

In Active Preparation.

Sixth year of publication. About 900 pp. Scarlet cloth, gilt lettered. Price 5s. A most complete guide to British, Colonial, Indian, and American Hospitals, Dispensaries, Nursing and Convalescent Institutions, and Asylums. A few copies of the "Annual" for 1894 may still be obtained on application to the Publishers, The Scientific Press (Limited), 428, Strand, London, W.C.

**NOTICE.—The Index for Vol. XVI. (ending September 24th, 1894) is on sale at the Office of this Journal, Price 2½d., post free.**

### SCRAPS AND GLEANINGS.

The Deaconess Hospital at Cleveland, Ohio, was burnt last week, with the loss of four lives.

Over £550 increase is shown in the year's Hospital Fund as collected by the working people of Leeds.

The annual children's ball at Nottingham, in aid of the funds of the children's hospital of that locality, has taken place.

The Faculté de Médecine at Paris comprises 1,002 foreign and 4,142 native students; 125 of these students are women, 26 being French.

In spite of efforts to collect sufficient subscriptions, the Newcastle Dispensary shows a very deficient balance-sheet in their annual report.

The trustees of the Chelsea Hospital for Women have just received another munificent gift (£500) from a generous donor who does not wish his name to transpire.

It is gratifying to notice that in spite of depression in trade at Glasgow during 1894, as well as the colliers' strike, the contributions to the Local Eye Infirmary have been well maintained in the aggregate.

Glasgow is threatened with an epidemic of small-pox, which, it is reported, has been started by an importation of rags from Ireland. More than thirty-six cases have been admitted into the hospital.

The Earl of Mar and Kellie has intimated his willingness to provide the hospital committee with a free site for the new accident hospital proposed to be erected by Miss Forrester Paton, Marshill House.

The Children's Infirmary at Liverpool presents a creditable report this year. The Convalescent Fund continues to be of much benefit, and seventy patients have been sent to West Kirby during the last twelve months.

A successful conversazione in aid of the funds of the Ballymena Cottage Hospital took place last month in the Ballymena (Belfast) Protestant Hall, consisting of tableaux vivants and vocal and instrumental music.

An English contemporary gives the actual statistics concerning Dr. Miles' examination of the eyesight of 4,000 persons in Connecticut. A percentage of 65 require glasses, he asserts. The period during which most touble is found with the eyes is between 20 and 30.

The highest death-rate in any town is to be found in the city of Mexico. This shows 40 per 1,000. The city is 7,000 above the sea level, but is defectively drained, which defect is perhaps the principal factor at work to account for the extraordinary death-rate.

Plans for the much required new premises for the Newcastle Throat and Ear Hospital have been submitted to the committee of the existing institution by Mr. Parsons. The project will involve an outlay of £2,000, which money, unfortunately, so far, is not forthcoming.

Female medical enterprise is advancing more quickly in America than anywhere else. The College of Physicians and Surgeons at Kansas City has now a lady professor, and Michigan University will soon have one, as an endowment fund for the purpose is receiving large contributions.

The Committee of Management of the Norwich Hospital Sunday and Saturday Fund regret that their report for 1894 must be a reflex of the depressed condition of surrounding circumstances. The collection day had been, unfortunately, wet and stormy, and £100 less had been received than in previous years.

A handsome banner has been offered to the committee of the Swansea General Hospital by Mr. Henry Studt. This, it is conjectured, will be very serviceable at large gatherings—Hospital Saturdays and the like. Mr. Studt has further promised to benefit the hospital by a monster gala and fête, such as he inaugurated last year for this much deserving charity.

Another instance of Mr. Passmore Edwards' generosity has been presented to us. He has now promised, we learn, to provide a cottage ospital for Liskeard; this, under certain conditions, will be established entirely at Mr. Edwards' own expense. Mr. Marshall has given a piece of land, and in all probability the work of erection will shortly be commenced.

A new and excellent resolution of the Aberdeen Royal Infirmary authorities declares that churches or parishes shall have the privilege of naming an infirmary bed for every £40 they send to the funds any one year, and that the bed thus set apart for the contributing year shall bear testimony on a printed card that the said bed represents the particular charity.

Whilst gratefully acknowledging the very liberal donation from the Hospital Saturday Fund of £250, the committee of the Brighton, Hove, and Preston Dispensary do not regard the increase in this year's liberality as a compensation for the loss of annual subscribers who supplied the funds upon which the committee felt they could depend as a permanent source of income.

The "electrophone" is apparently to become a real boon to mankind. We now learn that special arrangements will be made whereby patients in convalescent wards of hospitals will be able to enjoy through this means operas and concerts. The promoters of this most excellent scheme assure us in their prospectus that the electrophone will be supplied to all institutions with charity as the basis.

A special meeting of governors of the Royal Victoria Hospital, Bournemouth, has just been held at the Havergal Hall. The meeting was summoned to consider the desirability of abolishing the radius for admission to in-patients, the effect of which, if carried, would be to make the beds entirely free, except for domestic servants in service, and cases admitted by special payment. The resolution was put and carried *nem. con.*

### THE STUDENT OF MEDICINE.

### APPOINTMENTS.

A. Auld, M.B., C.M.Glasg., reappointed Medical Officer of Health for the No. 3 District of the Pontefract Union. W. Bassett, L.R.C.P.Lond., M.R.C.S.Eng., appointed Medical Officer for the Newport Branch of the South Wales Clerks' Association. A. Butler, L.R.C.S., L.R.C.P., appointed Medical Officer to the Walsham-le-Willows District of the Stow Union. C. M. Coates, L.R.C.P., L.R.C.S.Edin., appointed Medical Officer of the Creech St. Michael District of the Taunton Union. J. Currie, M.B.Lond., appointed House Surgeon to the Beckett Hospital and Dispensary, Barnsley. F. W. Gunn, M.D., B.S., L.S.Sc.Durham, M.R.C.S., L.R.C.P., L.S.A., A.K.C., appointed Medical Officer to the No 4 District, Morpeth Union. L. J. Hobson, M.D.Lond., B.S., F.R.C.S.Eng., Harrogate, reappointed Honorary Consulting Physician to the Royal Bath Hospital and Rawson Convalescent Home, Harrogate. A. M. Hynes, M.R.C.S. Eng., L.R.C.P.Edin., appointed Surgeon to Collingwood Dock, Bridewell, Liverpool. H. C. Linden, L.R.C.P.Edin., L.F.P.S. Glasg., appointed Medical Officer for the Harptree (No. 3) District of the Clutton Union. Mr. L. Lowe appointed Second Assistant to the Medical Officer of the Workhouse and Infirmary of the Parish of Paddington. W. R. Reith, M.B., appointed Assistant Medical Officer for the Workhouse of the Aston Union. C. G. Roberts, M.A.Cantab., M.B., B.C., reappointed Medical Officer of Health to the Halstead Urban Sanitary District. Mr. F. Watts appointed Medical Officer for the No. 3 District of the Yeovil Union. F. P. Weber, M.D., M.R.C.P.Lond., appointed Physician to the German Hospital, Dalston. Dr. Williamson appointed Medical Officer for the Northern Division of the Lancaster Union. P. M. Yearsley, F.R.C.S.Eng., appointed Clinical Assistant to the Ear Department of the Westminster Hospital. J. R. Ambler, M.R.C.S., appointed Assistant Medical Officer, Lunatic Asylum, Isle of Man.

### VACANCIES.

### Resident Medical Officers.

Central London Ophthalmic Hospital, 238A, Gray's Inn Road, W.C.—House Surgeon. Applications to the Secretary by February 12th.

Chelsea Hospital for Women, Fulham Road.—Resident Medical Officer; doubly qualified. Appointment for twelve months. Salary £60 per per annum, with board, lodging, and washing. Anæsthetist; medical women are eligible for this post. A small honorarium is given. Applications, on forms to be obtained at the hospital, to be sent to the Secretary by February 11th.

North-Eastern Hospital for Children, Hackney Road, Shoreditch, N.E.—Junior House Physician; doubly qualified. Appointment for six months. No salary, but board and lodging (including washing) provided. Applications to the Secretary, 27, Clement's Lane, E.C., by February 13th.

Ratcliffe Infirmary, Oxford. — House Surgeon; doubly qualified. Appointment for six months. Salary at the rate of £80 a year, with board, lodging, and washing. Applications on printed forms, to be obtained from the Secretary, to be sent to the Secretary by February 15th.

Salop Infirmary, Shrewsbury.—House Surgeon. Salary £100 per annum, with board and residence. Applications to the Board of Directors by February 11th.

THE STUDENT OF MEDICINE—*continued.*

**Non-Resident Medical Officers.**

Derbyshire Royal Infirmary, Derby.—Honorary Physician, Honorary Surgeon, Honorary Consulting Dental Surgeon. Applications to Walter G. Carnt, Secretary, by February 18th.
Kent and Canterbury Hospital, Canterbury.—Surgeon. Applications to the Secretary by February 19th.
Taunton and Somerset Hospital.—Honorary Surgeon. Must reside within two miles of the hospital. Applications to J. H. Biddulph Pinchard, Secretary, 13, Hammet Street, Taunton, by February 16th.

**EDINBURGH TRIPLE QUALIFICATION.**

FIRST EXAMINATION OF FOURTH AND FIFTH YEAR'S COURSE.

CHEMISTRY.

(Three questions to be answered.)

1. How is ammonia obtained from gas liquor? Describe the action of an aqueous solution of ammonia on an aqueous solution of: (*a*) Mercuric chloride; (*b*) aluminic sulphate; (*c*) zinc sulphate; (*d*) tartar emetic.

2. Describe the process by which lead and silver are obtained from argentiferous galena.

3. Show what occurs when the following substances are placed in water, and give the equation in each case: (*a*) Antimony trichloride; (*b*) bismuthic nitrate; (*c*) baric sulphide.

4. What is the chemical composition of gun-cotton? How is gun-cotton prepared? What are its properties? Give the formulæ of two analogous compounds.

ELEMENTARY ANATOMY.

(All the questions to be answered.)

1. Name the foramina and canals in the sphenoid bone, and state their relative position.
2. Describe the origin, relations, and nerve supply of the triceps muscle.
3. Describe the relations, attachments, and functions of the crucial ligaments of the knee joint.
4. Describe the dissection necessary to expose the posterior tibial artery. Name its branches.

ELEMENTARY BIOLOGY.

(All the questions to be answered.)

1. Where is starch for the most part formed within a plant? What must happen to it before it can pass from one part of a plant to another? What analagous process occurs in animals?
2. Mention in order the parts of a pigeon's alimentary canal and state their uses.
3. Describe a cross section of a young rose-stem.
4. How does the skeleton of a lobster or crayfish differ from that of a bird: (*a*) as to position; (*b*) as to origin; (*c*) as to the material of which it is composed?

PHYSICS.

(All the questions to be answered.)

1. Give a short account of the process called osmosis.
2. If the acceleration due to gravity be 32 feet per second per second, how far does a stone fall in 3½ seconds?
3. Describe the process called convection of heat, and give some examples of it.
4. State how you would magnetise temporarily a rod of iron such as a poker, first without, and secondly with, the use of a voltaic current.

